# Prosthetic Tricuspid Valve Thrombosis

**DOI:** 10.7759/cureus.2928

**Published:** 2018-07-05

**Authors:** Ahmed Zaghloul, Corina Iorgoveanu, Aakash Desai, Molly Silkowski, Kathir Balakumaran

**Affiliations:** 1 Internal Medicine, University of Connecticut Health Center, Farmington, USA; 2 Internal Medicine, Ohio State University, Ohio, USA; 3 Cardiology, University of Connecticut Health Center, Farmington, USA

**Keywords:** tricuspid valve, thrombosis, valve thrombosis, prosthetic valves

## Abstract

Prosthetic valve thrombosis, a serious complication of prosthetic valve replacement, can be lethal without proper treatment. Right-sided valve thrombosis is rare but several therapeutic modalities can be considered: anticoagulation therapy, fibrinolysis, or surgery. Here, we report a case of significant tricuspid valve thrombosis which failed fibrinolytic therapy requiring repeat sternotomy with repeat tricuspid valve replacement with a porcine bioprosthesis.

## Introduction

Prosthetic valve thrombosis is a rare, but serious complication that can become lethal if not treated properly [1]. Right-sided valve thrombosis is rare but several therapeutic modalities can be considered: anticoagulation therapy, fibrinolysis, or surgery.

## Case presentation

**Figure 1:**
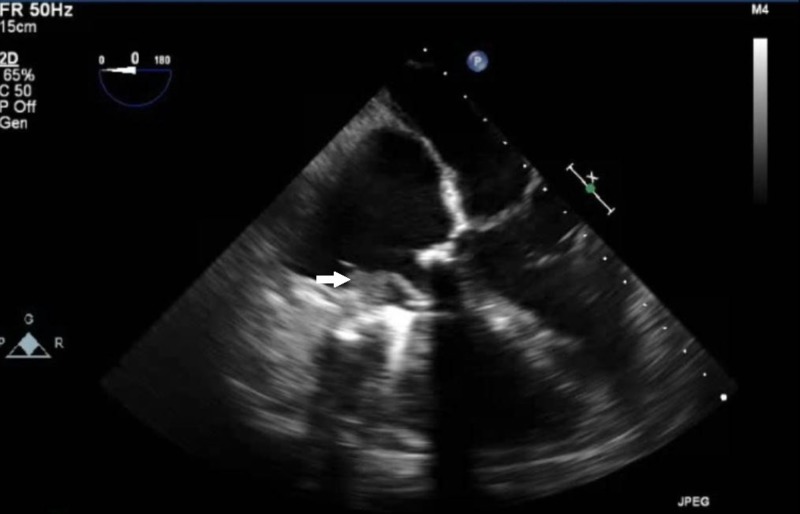
Transesophageal echocardiogram view of the tricuspid valve with evidence of a large thrombus (arrow) along the atrial aspect of the mechanical tricuspid valve

**Figure 2:**
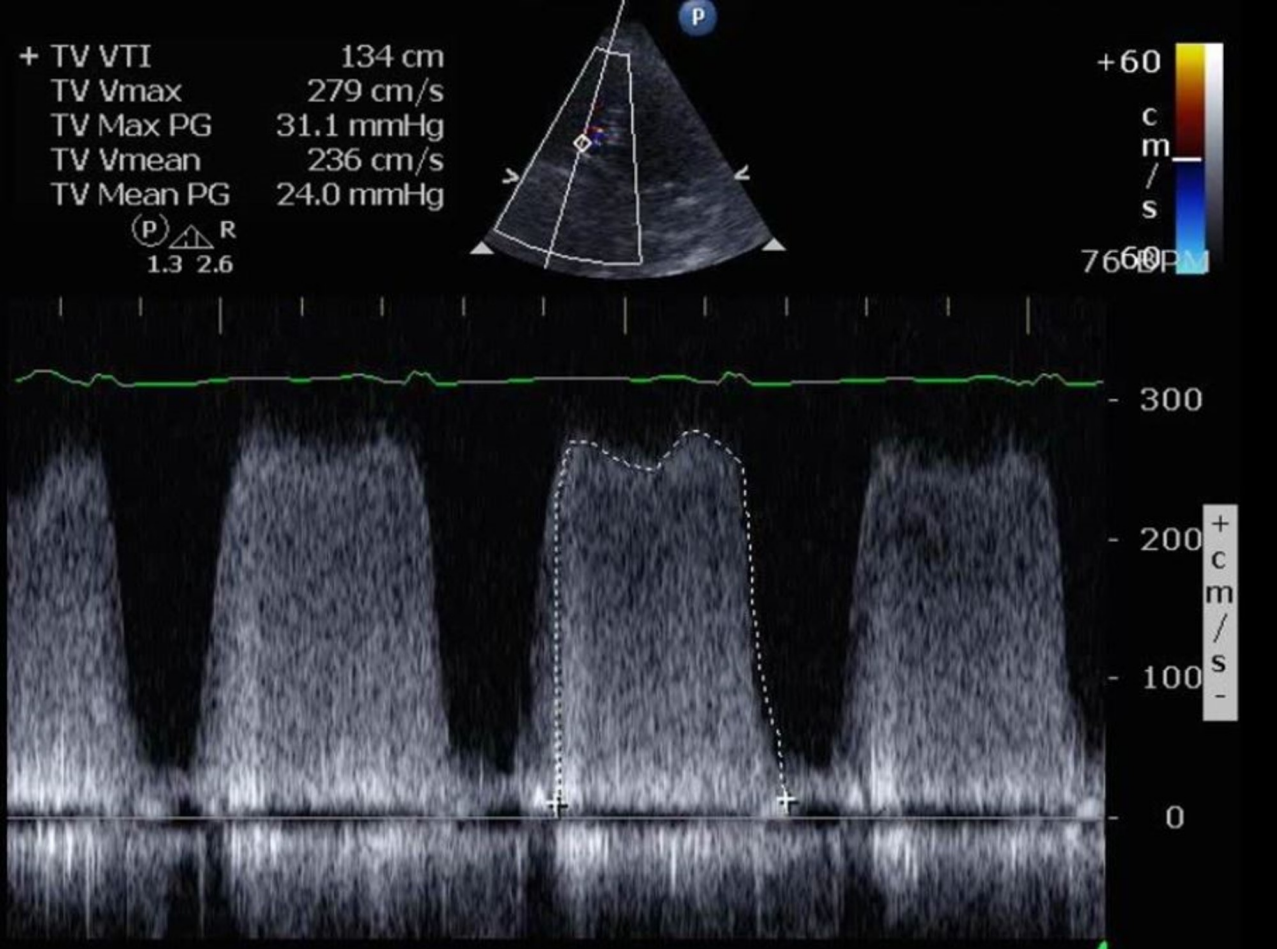
Continuous wave Doppler across the tricuspid valve; mean gradient is 24 mmHg

**Figure 3:**
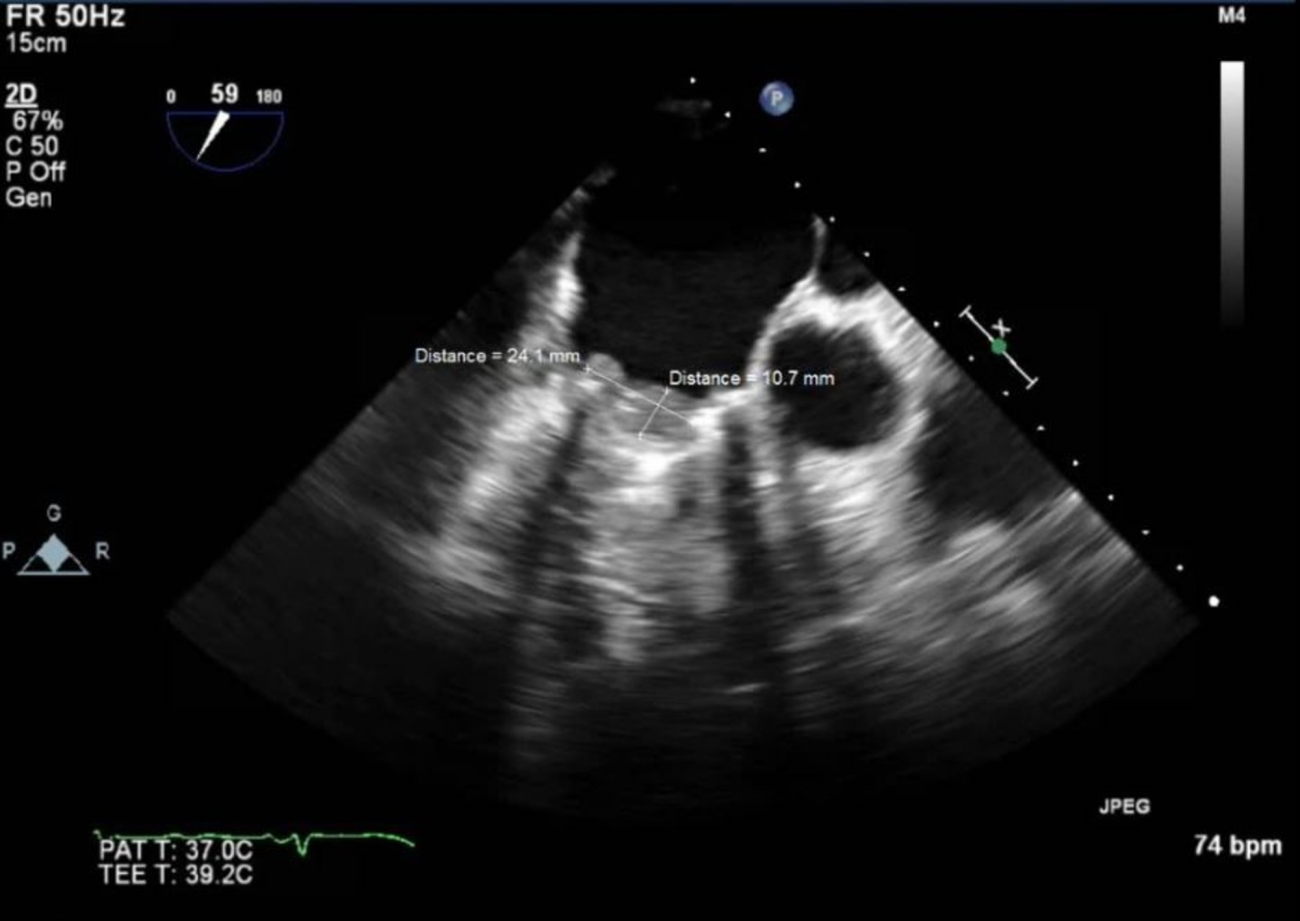
Transesophageal echocardiogram view of the tricuspid valve revealing a thrombus measuring 2.41 cm x 1.07 cm along the atrial aspect of the mechanical tricuspid valve

A 42-year-old female with a history of morbid obesity and tricuspid valve endocarditis with mechanical TV replacement, who had stopped taking Coumadin for one week, presented with worsening dyspnea, facial cyanosis, marked lower extremity edema, and increased abdominal girth. Transthoracic echocardiography (TTE) and transesophageal echocardiography (TEE) revealed significant tricuspid valve thrombosis with severely immobile leaflets and severe pulmonary hypertension (Figures 1-3).

**Figure 4:**
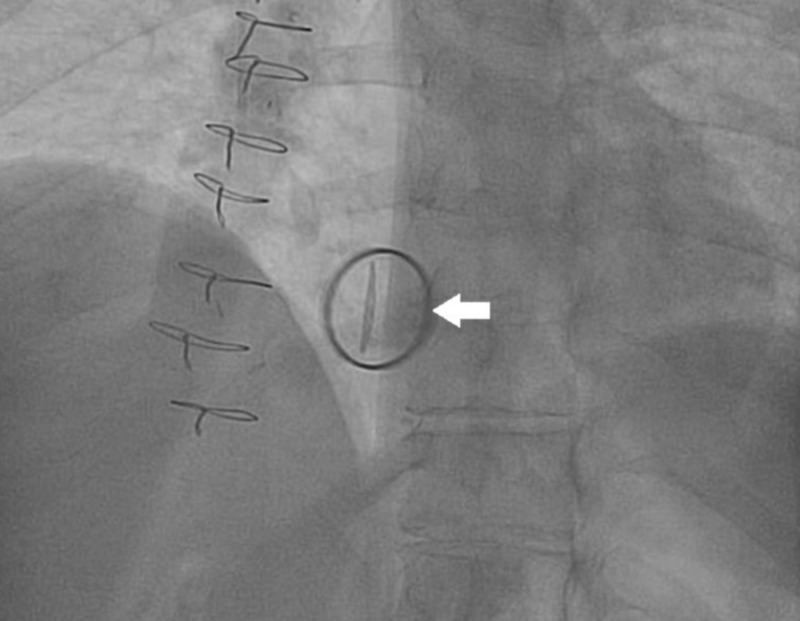
Fluoroscopic view of the mechanical prosthetic tricuspid valve with immobile leaflets (arrow) secondary to extensive thrombus formation

She was initially started on a high-intensity heparin infusion with 8000U bolus followed by 1800U per hour infusion. She was brought to the interventional laboratory where a 7-French triple-lumen central venous catheter was advanced to the right atrium via the right internal jugular vein under fluoroscopic guidance. A bolus of 2 mg of alteplase (tPA) was delivered followed by a continuous infusion of 1 mg/hour for 24 hours. Fluoroscopy revealed a tricuspid valve with severely reduced mobility (Figure 4).

The gradient across the tricuspid valve was above 20 mmHg and right atrial pressure was elevated at 22 mmHg. Repeat gradients were assessed 24 hours later, and TV gradient remained elevated above 20 mmHg, with severely elevated central venous pressure, and markedly reduced cardiac index. The patient underwent redo sternotomy with redo tricuspid valve replacement with a porcine bioprosthesis. Her postoperative course was uneventful. She was started on coumadin and bridged with heparin infusion and was discharged home with INR 2.6.

## Discussion

Prosthetic valve thrombosis is a rare but serious complication of prosthetic valve replacement that can be lethal if not treated properly. Overall, the incidence of thrombosis is reported to be between 0.1% and 5.7% per year, with 0.5% to 6% of thromboses involving the aortic or mitral valvular positions and up to 20% in the tricuspid valve [1-2]. The risk of tricuspid prosthetic valve thromboses is believed to be higher than that of the aortic or mitral due to the fact that the lower pressures on the right side of the heart lead to slower blood flow across the valve. Moreover, the risk of thromboses in prosthetic valves is increased in patients in prothrombotic states such as pregnancy, atrial fibrillation or obesity. Additionally, it is believed that pulmonary hypertension can lead to endothelial injury in the right ventricle and atrium, leading to an increased susceptibility to thrombus formation.

The New York Heart Association (NYHA) has classified prosthetic valvular heart disease (PVHT) in functional classes I to IV. Classes I and II are non-obstructive forms of PVHT, an incidental echocardiographic finding in patients with evidence of thromboembolic stroke or peripheral arterial embolism. Those classified in NYHA functional classes III or IV correspond to obstructive forms of PVHT. Obstructive forms of PVHT are those with obvious hemodynamic repercussions potentially resulting in cardiogenic shock and cerebral or peripheral emboli. Patients with obstructive tricuspid valve thrombosis usually present with signs of right heart failure such as peripheral edema, ascites, and an audible prosthetic click during auscultation. Because of our patient’s shortness of breath, she was classified as NYHA Class III [3].

Diagnosis of PHVT is usually confirmed by TTE, TEE, or cine fluoroscopy. TTE is often performed first, though acoustic shadowing and artifacts created by mechanical prosthetic valves may limit its usefulness in evaluating valve motion. Doppler parameters can be utilized to assess prosthetic valve dysfunction. According to the American Society of Echocardiography, obstruction of a prosthetic tricuspid valve is suggested by continuous wave Doppler with an emptying velocity greater than 1.7 m/s, mean gradient greater than 6 mmHg, or pressure half time greater than 230 ms [4].

Once the diagnosis of PHVT is confirmed, several therapeutic modalities can be considered: anticoagulation therapy, fibrinolysis, or surgery. The traditional treatment for PVHT has been an emergent or urgent surgery, with the replacement of the prosthesis being the most widely used approach. However, mortality from surgery can be as high as 69% depending on the NHYA functional class group [3]. This high mortality rate with the surgery has stimulated the development of alternative therapeutic approaches over the years including thrombolysis which has become an appropriate option in certain cases.

According to the guidelines of the American Heart Association/ American College of Cardiology (AHA/ACC) and the American College of Chest Physicians (ACCP), in contrast to left-sided PVHT, thrombolytic therapy is reasonable for right-sided PVHT with NYHA functional class III-IV symptoms or clot burden [3,5-7].

In support of this statement, many studies found the benefit of thrombolytics for right-sided valve thrombosis. In one retrospective study, 16 patients underwent thrombolytics for right-sided mechanical valves, eight for pulmonic valves and the other eight for tricuspid. For the tricuspid valve patients, 75% had a successful response to thrombolytic therapy with an in-hospital survival rate of 87.5% [8]. In a meta-analysis by Rose et. al. from 2002, thrombolytic therapy showed superiority over surgical embolectomy (OR for mortality: 2.83, 95% CI 1.04–7.69) and anticoagulation (OR for mortality: 3.03, 95% CI 1.02–3.125) [9]. Furthermore, a meta-analysis by Castilho et al. showed lower mortality in patients treated with thrombolytic therapy and that the rate of embolic events in the thrombolytic group was higher than in patients treated by surgery. Compared to mitral valve thrombosis, fibrinolytic agents for prosthetic tricuspid valve thrombosis have been associated with a high success rate and low complication rate as there is no risk of cerebral embolism and the incidence of thromboembolism to the lungs is usually less serious than a cerebrovascular episode.

The most commonly used thrombolytic agent for this therapeutic approach is streptokinase, though urokinase and alteplase (t-PA) have also been successfully used. Choosing the correct thrombolytic can depend on several factors including cost (streptokinase has lower cost), time to attain maximal pharmacological effect (t-PA is more rapid), half-life of thrombolytic agent- an important issue if urgent surgery might be required due to failure of thrombolytics (t-PA has a more rapid reversal) and hemorrhagic complications (Streptokinase and urokinase have lower cerebral hemorrhage rates compared to t-PA) [4].

Concerning the mode of administration of therapy, there is limited evidence to prove whether systemic or catheter-based thrombolytics are the more effective method. So far, there is one reported case of a successfully treated thrombosed tricuspid valve with catheter-based thrombolytics [10]. Zhang et al. [10] described a 32-year-old female with an extensive cardiac history due to Ebstein’s anomaly who received a mechanical tricuspid valve. The patient was found to have a tricuspid thrombus with a valve gradient of 9 mmHg. She proceeded to undergo catheter-based thrombolytic therapy over the span of two days. Her valve gradient was successfully reduced from 9 mm Hg to 4mm Hg at the end of day one, and by the end of day two, it was reduced further to 2 mm Hg with the administration of t-PA.

Although in our patient, catheter-based thrombolytics were used without success, it is possible that the rate of infusion was not rapid enough as no bolus was given on the first day. Furthermore, the successful catheter-based patient was hemodynamically stable and able to receive a succeeding day of thrombolytics, whereas our patient continued to decline hemodynamically, warranting surgery.

The other treatment options for PVHT are surgery and anticoagulation. Although surgery has been the traditional treatment, it should be reserved for cases with a pannus, thrombolytic failure, or contraindication to thrombolysis such as hemorrhagic diabetic retinopathy, active internal hemorrhage, history of hemorrhagic stroke, brain tumor, recent traumatic brain injury, or blood pressure over 200/120 mmHg [4]. Anticoagulation, the other option for PVHT therapy, has also been successfully implemented, but its greatest efficacy is among patients that are asymptomatic (class NHYA I/II) or when used in conjunction with thrombolytics [8].

## Conclusions

As the sole therapy, anticoagulation will not treat a symptomatic thrombus. Because our patient was hemodynamically unstable after the combination of thrombolytics and anticoagulation, she underwent successful surgery with a new bioprosthetic valve. In conclusion, our case demonstrates the typical presentation of prosthetic tricuspid valve thrombus and the value of thrombolytics, anticoagulation, and surgery in treatment.
